# Supplementary material for: Increased L-Selectin on Monocytes Is Linked to the Autoantibody Profile in Systemic Sclerosis
Source: Int J Mol Sci. 2022 Feb 17;23(4):2233. doi: 10.3390/ijms23042233 (PMC8880182; doi:10.3390/ijms23042233)
Supplement: Supplementary file 1 [file ijms-23-02233-s001.zip › ijms-1600850-supplementary.pdf]

Table S1. Additional clinical and laboratory data of SSc patients.

**Variable**	
**Pulmonary function tests**	
DLCO (%)	66 (32)
FEV1 (%)	92 (27)
**Blood tests**	
Leukocytes (× 10^9/L)	6 (2)
Thrombocytes (× 10^9/L)	234 (115)
Haemoglobin (g/L)	133 (17)
ESR (mm/h)	18 (26)
SAA (mg/L)	6 (14)

All values are presented as median with interquartile range. DLCO, diffusing capacity for carbon monoxide; ESR, erythrocyte sedimentation rate; FEV1, forced expiratory volume in the first second; SAA.
